# Dimensional Analysis and Optimization of IsoTruss Structures with Outer Longitudinal Members in Uniaxial Compression

**DOI:** 10.3390/ma14082079

**Published:** 2021-04-20

**Authors:** Hanna B. Opdahl, David W. Jensen

**Affiliations:** 1 Department of Civil Engineering, Brigham Young University, Provo, UT 84602, USA; 2 Jensen Technologies, LLC, 525 W 3050 S, Mapleton, UT 84664, USA

**Keywords:** IsoTruss structures, lattice structures, buckling, dimensional analysis, mass minimization

## Abstract

This study analyzes the buckling behavior of 8-node IsoTruss^®^ structures with outer longitudinal members. IsoTruss structures are light-weight composite lattice columns with diverse structural applications, including the potential to replace rebar cages in reinforced concrete. In the current work, finite element analyses are used to predict the critical buckling loads of structures with various dimensions. A dimensional analysis is performed by: deriving non-dimensional Π variables using Buckingham’s Π Theorem; plotting the Π variables with respect to critical buckling loads to characterize trends between design parameters and buckling capacity; evaluating the performance of the outer longitudinal configuration with respect to the traditional, internal longitudinal configuration possessing the same bay length, outer diameter, longitudinal radius, helical radius, and mass. The dimensional analysis demonstrates that the buckling capacity of the inner configuration exceeds that of the equivalent outer longitudinal structure for the dimensions that are fixed and tested herein. A gradient-based optimization analysis is performed to minimize the mass of both configurations subject to equivalent load criteria. The optimized outer configuration has about 10.5% less mass than the inner configuration by reducing the outer diameter whilst maintaining the same global moment of inertia.

## 1. Introduction

Composite lattice trusses are high strength, lightweight structures that are being developed and implemented in disciplines including aerospace structures, automotive bodies, and civil infrastructure [1,2,3]. In addition to an excellent strength-to-weight ratio, these structures demonstrate substantial damping, stiffness, flexural capacity, and corrosion resistance [4]. Possessing adaptable geometries, these structures can be reconfigured to serve as beams, struts, columns, shells, and the cores of sandwich composites [5].

IsoTruss^®^ structures are a distinct variation of open-lattice composite grid columns. The general structure is comprised of longitudinal and helical members that are aligned with anticipated load criteria to maximize strength-to-weight [6]. Longitudinal members are straight, continuous members that span the overall length, whereas helical members wind piece-wise linear around the structure to form a continuous helical-like member. All members are made of fiber tows encased in resin, and consolidated with external wrapping techniques such as braided sleeves, coiled sleeves, Kevlar wrapped sleeves, or polyester shrink-tape sleeves [7]. Various fiber and resin constituents have been used, including graphite, fiber-glass, and basalt tows with diverse epoxy resins [8]. Structural properties such as the number of nodes (i.e., the number of longitudinal members), the number of carbon tows in each member, and the materials are selected according to the distinct design criteria.

The structural performance of composite grid columns, including IsoTruss structures, has been widely studied to identify and understand the governing failure modes. Loaded in axial compression, these columns generally fail in material failure, global buckling, local buckling modes, and strut crushing [9,10,11]. Buckling is a prevalent failure mode that has been studied using experimental, numerical, analytical, and optimization methods.

Finite element (FE) methods are a prevalent numerical approach that is broadly used to assess and compare the structural proficiency of diverse configurations with various material properties [12,13,14,15,16]. Buckling models of composite structures have been developed within FE applications to capture both the linear and nonlinear modes. Linear eigenvalue buckling models are used to predict critical buckling loads of global and localized buckling [17,18]. Nonlinear models are enhancing the fidelity of buckling analyses, facilitating greater understanding of post-buckling capacities and the influence of shear deformations [19,20,21,22].

Analytical methods such as mathematical expressions are often used to verify experimental data and validate results predicted by numerical models. While the fidelity of these expressions are limited by the corresponding assumptions, the expressions provide a baseline to characterize interrelations between design parameters (e.g., material properties and structural geometry) and performance criteria (e.g., ultimate capacity or structural efficiency) [23,24]. Such expressions have been derived for composite structures using traditional mechanics principles including strain energy formulation or classical laminate theory, and are being augmented to account for transverse curvature and individual member strains [25,26,27].

Optimization methods are often used in the preliminary design phase of composite structures to maximize strength-to-weight and other desirable characteristics [28,29,30]. The optimization objectives and constraints are defined with various methods, including the use of analytical expressions that demonstrate sufficient fidelity [23,26,31]. Both gradient-free and gradient-based frameworks have been employed in preceding studies to maximize structural efficiency. Gradient-free methods, such as the non-sorting genetic algorithm II (NSGA-II), are used frequently to optimize structural configurations and facilitate multi- or single-objective optimization of both discrete and continuous design variables [32,33]. Gradient-based methods are used in other studies to perform sensitivity analyses in addition to mass minimization [26,31].

In preceding research studies, many configurations of IsoTruss structures with inner longitudinal members have been analyzed by manufacturing experimental specimens and performing physical testing [34,35]. Implementing numerical methods such as FE analysis and optimization studies has expedited the design process, facilitating the preliminary assessment of alternative configurations [24,26,36,37,38,39]. This study is part of a broad research initiative to develop and implement numerical and optimization methods for the preliminary design of IsoTruss structures. The following studies by Opdahl et al. preceded the current work to develop numerical techniques for analyzing IsoTruss structures with inner longitudinal members: a linear eigenvalue buckling FE model was validated with experimental testing and verified with analytical expressions [24]; an analytical expression was derived to predict the local/shell-like buckling mode [26]; trends between design parameters (i.e., outer radius, radius of longitudinal members, radius of helical members, and bay length) and the shell-like buckling mode were characterized in a dimensional analysis [39]; the mass of an inner longitudinal configuration was minimized in an optimization study using both gradient-based and gradient-free optimization algorithms [26].

**Figure 1:**
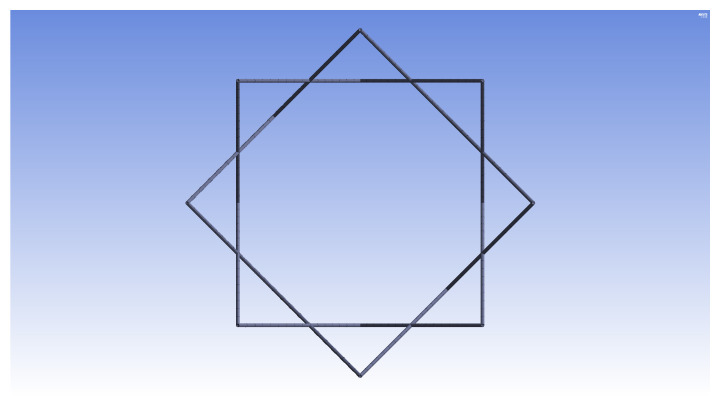
End view of IsoTruss structure [26].

**Figure 2:**
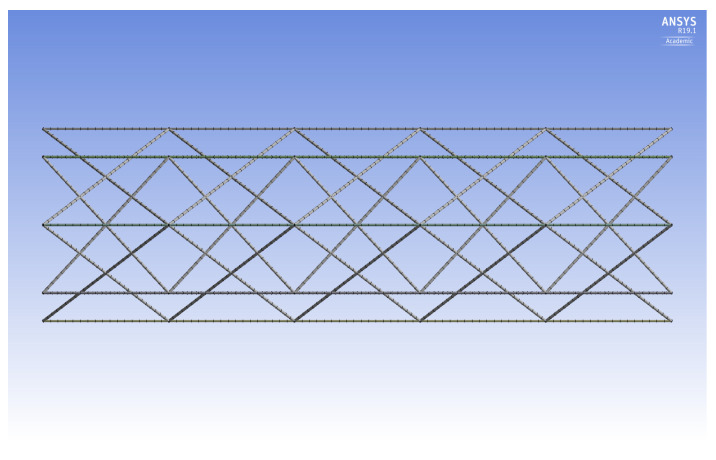
Side view of IsoTruss structure with outer longitudinal members (i.e., OLC) [26].

The purpose of the current study is to adapt the aforementioned numerical, dimensional, and optimization methods (developed for inner longitudinal configurations [26]) to the design of IsoTruss structures with outer longitudinal members. The outer longitudinal configuration (OLC) possesses the same geometric characteristics as the inner longitudinal configuration (ILC) except that the longitudinal members are placed at the outer diameter of the structure, spanning between the nodes. Figure 1 is the end view of an IsoTruss structure. A side view of the OLC is shown in Figure 2. Refer to the works presented by Kesler and Opdahl for more explanation of IsoTruss orientation and geometry [26,40].

OLC and ILC structures of equal bay length, outer diameter, and member radii are equivalent in mass. By pushing the longitudinal members to the outer diameter, the global moment of inertia of the structure is increased without increasing the mass. Hence, the OLC is inherently more resistant to global buckling than the ILC of equal dimensions. On the other hand, the placement of the longitudinal members in the OLC increases the span of the longitudinal struts, thereby increasing the susceptibility to local buckling.

Due to inherent manufacturing complexity, experimental testing has not been widely performed on the OLC, therefore, there is limited physical data to demonstrate the structural performance and buckling behavior. The current study produces data from dimensional analysis (akin to that performed by Opdahl and Jensen [39]), FE modeling, and optimization techniques (based on the framework presented in [26]) to explore four subtopics. First, the data are used to characterize trends between the OLC design parameters and the buckling capacity. Second, FE predictions are plotted with analytical predictions to verify the accuracy of an analytical expression presented herein. Third, the relative performance of the OLC with respect to the ILC is analyzed via dimensional analysis. Finally, the OLC and ILC are optimized with respect to mass (via gradient-based techniques) to indicate the distinct advantages of each configuration under the same loading criteria.

## 2. Methods

Three methods of analysis are implemented in the current study to analyze the buckling behavior of the OLC and compare its relative performance to the ILC. First, a dimensional analysis is performed to characterize the interrelations between the governing design parameters and the critical buckling load. The parameters are reduced to three non-dimensional independent Π variables via Buckingham’s Π Theorem (BPT). Likewise, the critical buckling load is also reduced to a non-dimensional term via BPT. Next, FE methods are used to predict critical buckling loads for diverse structural configurations. FE analyses are performed in ANSYS WorkBench based on the validated methods discussed by Opdahl and Jensen [24]. The predictions are used to assess the relative accuracy of analytical expressions for local buckling in the OLC. Finally, the optimization techniques presented by Opdahl [26] are implemented to optimize the OLC and ILC with respect to mass. These methods are expounded in the subsequent sections.

### 2.1. Dimensional Analysis

#### 2.1.1. Buckingham’s Π Theorem

The governing design parameters of the OLC are the same as those identified by Opdahl and Jensen [39] to govern the buckling behavior of the ILC (i.e., longitudinal radius [$r_{L}$], helical radius [$r_{H}$], bay length [*b*], outer radius [*R*], and Young’s modulus [$E_{z}$]). Therefore, the three independent Π variables derived therein are used in the current study, and are provided in Equation (1) for reference. Kesler and Opdahl provide additional figures and description of these governing design parameters [26,40]. In the current study, both global and local buckling modes are considered, hence, the critical buckling load, $P_{cr}$, is selected as the dependent variable of interest in place of the shell-like buckling load used by Opdahl and Jensen. While the global length, *L*, is not explicitly defined as a design parameter in BPT, it is implicitly incorporated in the FE predictions of the critical buckling load of the global buckling mode.

(1) $$\begin{array}{cc} \Pi_{1}= & \frac{r_{L}}{R}\\ \Pi_{2}= & \frac{r_{H}}{R}\\ \Pi_{3}= & \frac{b}{R}\\ \Pi_{0}= & \frac{P_{cr}}{E_{z}\cdot R^{2}} \end{array}$$

#### 2.1.2. Trend Analysis

Trend analyses are performed for the OLC in the same manner as those presented by Opdahl and Jensen [39] for the ILC. That is, a trend analysis is performed for each independent Π variable with respect to the dependent Π variable. Each trend analysis consists of three sets of FE analyses, and each set of FE analyses has different design parameters to demonstrate how the interrelations may vary with respect to different geometric dimensions. Each set of geometric dimensions is distinguished by the ratios $\Pi_{3}$-to-$\Pi_{2}$, $\Pi_{3}$-to-$\Pi_{1}$, or $\Pi_{1}$-to-$\Pi_{2}$ for the trend analyses of variables $\Pi_{1}$, $\Pi_{2}$, and $\Pi_{3}$, respectively. The independent Π variables and the Π ratios of each FE set are presented in Table 1. The values of the variables were selected to provide Π ratios that are round numbers within the design space of the long, light-weight IsoTruss structures typical of the Rackliffe et al. [34] specimens.

Trend analyses are also used in the current study to compare the relative performance of the OLC and the ILC. A trend analysis is performed for each independent Π variable of the ILC configuration using the design parameters of Set 2. The results of each ILC analysis are plotted with the corresponding results of the Set 2 OLC analysis.

### 2.2. Finite Element Models

The FE analyses consist of static structural analyses and eigenvalue buckling analyses to predict the critical buckling load and mode of each distinct configuration. The boundary conditions were defined as fixed-free at the ends of the IsoTruss structure, and the compression load was defined as 500 N (112 lb.). The density of the FE mesh was 10 $m^{-1}$ (0.25 ${\mathrm{in}.}^{-1}$). The fixed design parameters that correspond with each set of trend analyses (i.e., number of tows per longitudinal or helical member [$N_{t}$], number of bays [$N_{b}$], and overall length [*L*]) are summarized in Table 2.

**Figure 3:**
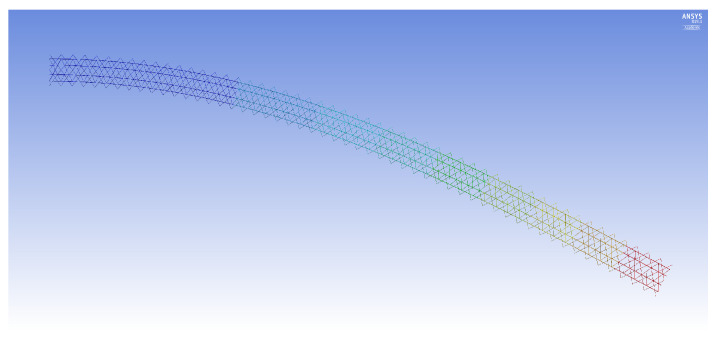
Global buckling of an inner longitudinal IsoTruss structure [26].

**Figure 4:**
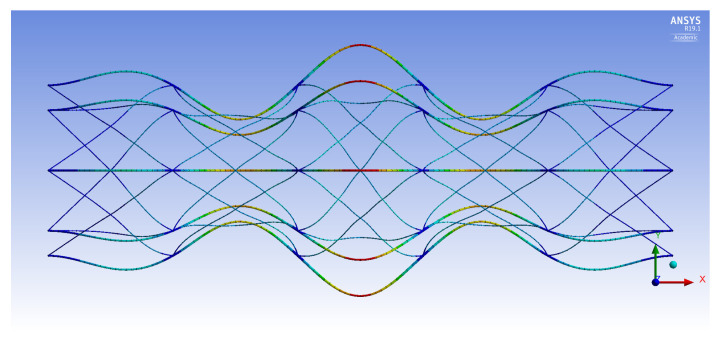
Local buckling of an outer longitudinal IsoTruss structure (side view) [26].

**Figure 5:**
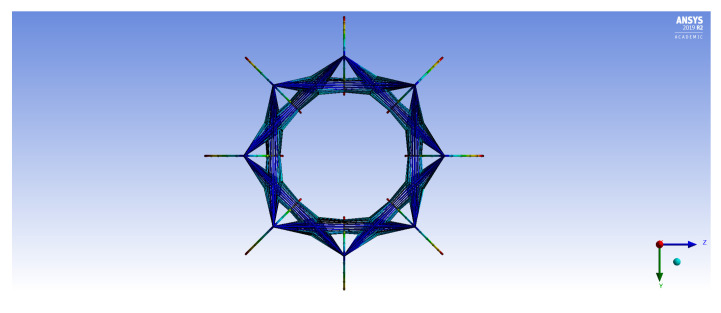
Local buckling of an outer longitudinal IsoTruss structure (end view) [26].

The FE models demonstrate two general buckling modes: global buckling and local buckling. The global buckling mode follows the typical model and expression of Euler-buckling of a cantilever column. Figure 3 shows the global buckling mode of an IsoTruss with inner longitudinal members, produced from an FE model. The local buckling mode occurs over the longitudinal members such that the struts buckle either inward or outward symmetrically with a wavelength of two bays. Figure 4 and Figure 5 demonstrate the local buckling mode of an outer longitudinal IsoTruss, shown from the side and end of the structure, respectively. The models have a mesh density of 200 $m^{-1}$ (5.08 ${\mathrm{in}.}^{-1}$).

### 2.3. Optimization Techniques

The gradient-based techniques presented by Opdahl are implemented in the current study to optimize the OLC with respect to the same bounds and constraints as those imposed on the ILC by Opdahl [26]. The code employs the built-in optimizer ‘fmincon’ to minimize mass using a gradient-based algorithm. The framework executes the optimization in two stages. First, the optimizer minimizes the mass, treating all design variables as continuous. Second, the discrete variables (i.e., the number of bays and the number of longitudinal tows) are rounded to integer values, fixed as input variables, and the outer diameter is re-optimized as a continuous variable. Algorithmic differentiation is implemented within the analysis to supply the gradients of the objective and constraint functions to the optimizer. The sensitivity derivatives and Lagrange multipliers are produced with the optimized solution.

The problem definition therein includes a constraint for the eigenvalue of the longitudinal strut buckling mode, $\lambda_{l}$, and shell-like buckling mode, $\lambda_{sb}$, that are typical for the ILC. Contrary to the shell-like buckling mode exhibited by the ILC, the local bay-level buckling of the OLC demonstrates complete radial symmetry, with the longitudinal struts all buckling either outward or inward at a given point along the longitudinal axis. The shell-like buckling equation for the ILC local buckling mode was replaced with an equation that predicts local buckling in the OLC. This local buckling mode, defined by the bay buckling load, $P_{b}$, is implemented to replace $P_{l}$ and $P_{sb}$ of the ILC. The analytical expression that is used to predict local buckling in the OLC is shown in Equation (2). The boundary constraints imposed by the helical struts are approximated as pinned joints with the effective length factor, $\mu_{b}$, of one.

(2) $$\begin{array}{cc} P_{b}= & \;N\cdot \frac{\pi^{2}\cdot E_{z}\cdot I_{L}}{{\left(\mu_{b}\cdot b\right)}^{2}}\\ \mu_{b}= & \;1.0\\ \lambda_{b}= & \;\frac{P_{b}}{P} \end{array}$$

While the constraining influence of the nodes on the local buckling mode is approximated in the current work as a pinned joint, other studies explore the boundary constraint as a function of design parameters such as member radius, material properties, and/or the inclination angle [34,36,41,42]. Opdahl documents the derivation of a boundary constraint coefficient, $\mu_{sb}$, for the ILC that is a function of the geometry and material of the longitudinal members [26]. As the stiffness of the longitudinal members increases, the rotational stiffness of the node increases, decreasing the validity of the pinned-joint assumption. The influence of the helical members at the nodes is expounded in the discussion section based on the results of the analyses performed herein. Additional exploration should be performed in a subsequent study to enhance the fidelity of the analytical expression shown in Equation (2).

The global buckling load, $P_{g}$, is predicted using the Euler-buckling equation for a cantilever column. The moment of inertia coefficient, *c*, is selected based on the derivation by Winkel [36] for outer longitudinal members (see Equation (3)).

(3) $$\begin{array}{cc} P_{g}= & \;\frac{\pi^{2}\cdot E_{z}\cdot I_{g}}{{\left(\mu_{g}\cdot L\right)}^{2}}\\ \mu_{g}= & \;2.0\;(\mathrm{for}\;\mathrm{fixed}-\mathrm{free}\;\mathrm{column})\\ I_{g}= & \;c\cdot A_{L}\cdot R^{2}\\ c= & \;4.0\;(\mathrm{for}\;8-\mathrm{node}\;\mathrm{IsoTruss}\;\mathrm{with}\;\mathrm{outer}\;\mathrm{longitudinal}\;\mathrm{members})\\ \lambda_{g}= & \;\frac{P_{g}}{P} \end{array}$$

The adjusted problem definition of the optimization analysis performed in the current study is summarized mathematically in Equation (4).

(4) $$\begin{array}{cc} \mathrm{Minimize} & \;M\\ \mathrm{with}\;\mathrm{respect}\;\mathrm{to} & \;N_{b},N_{t_{L}},D\\ \mathrm{subject}\;\mathrm{to}\; & \;1.0-\lambda_{g}<0\\ \; & \;1.0-\lambda_{b}<0\\ \; & \;\sigma -\sigma_{u}<0\\ \; & \;\left[N_{b}\;N_{t_{L}}\;D\right]>\left[20\;9\;4\right]\\ \; & \;\left[N_{b}\;N_{t_{L}}\;D\right]<\left[100\;13\;8\right]\\ \; \end{array}$$

## 3. Results

The results from the FE analyses and analytical predictions are presented as four subtopics in the subsequent sections. The first two subtopics focus on characterizing the buckling behavior of the OLC. First, the FE analyses of the OLC are used in trend analyses to assess the interrelations between each independent Π variable and the dependent Π variable. Second, the analytical predictions of the OLC critical buckling loads are plotted with the FE predictions. The plots indicate the extent to which the analytical expression adequately predicts critical buckling with respect to the FE results. The next two subtopics compare the performance of the OLC with that of the ILC. First, data collected for the OLC and ILC trend analyses are plotted together to indicate the relative performance of the configurations within the design space of the trend analyses. Second, the analytical expression for bay-level buckling in the OLC is implemented in the gradient-based optimization routine presented by Opdahl [26] to compare the OLC and ILC structures that are optimized for mass.

### 3.1. Trend Analyses of OLC

**Figure 6:**
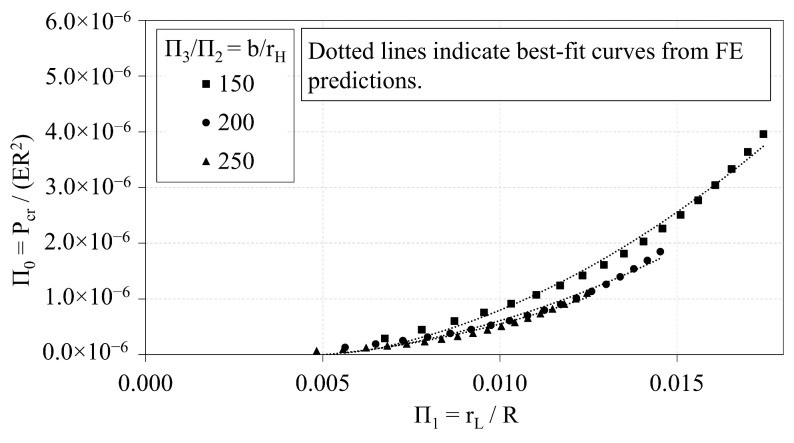
OLC $\Pi_{0}$ vs. $\Pi_{1}$ [26].

**Figure 7:**
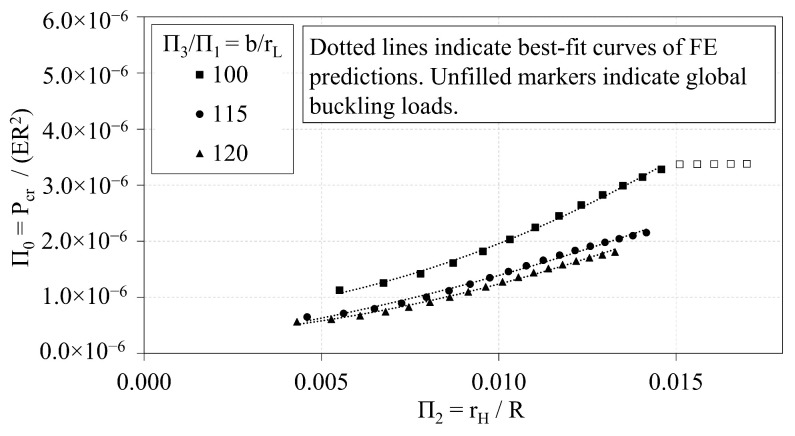
OLC $\Pi_{0}$ vs. $\Pi_{2}$ [26].

**Figure 8:**
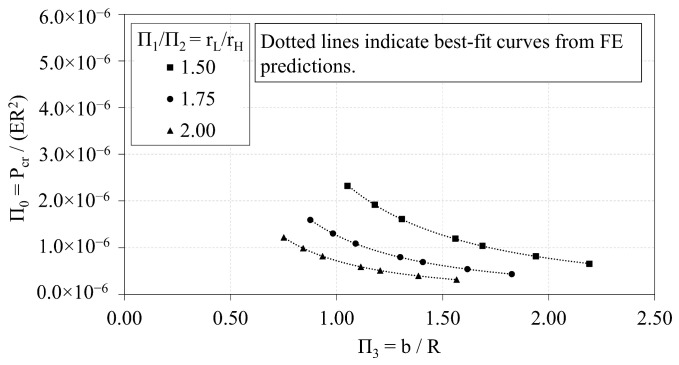
OLC $\Pi_{0}$ vs. $\Pi_{3}$ [26].

Data from the OLC trend analyses are first used to characterize trends between the non-dimensional, independent Π variables. The independent Π variables $\Pi_{1}$, $\Pi_{2}$, and $\Pi_{3}$ are plotted against $\Pi_{0}$ in Figure 6, Figure 7 and Figure 8, respectively. Local buckling loads are represented in the plots by solid markers, whereas global buckling loads are represented by markers that are unfilled. The dotted lines represent the best-fit curves.

Figure 6 indicates that increasing $\Pi_{1}$ induces a quadratic increase in $\Pi_{0}$. It follows that increasing the radius of the longitudinal members induces a quadratic increase in the critical buckling load. Figure 6 also indicates that the $\Pi_{0}$ vs. $\Pi_{1}$ curve shifts downward as the ratio of *b*-to-$r_{H}$ increases. The general quadratic expression that relates $\Pi_{1}$ to $\Pi_{0}$ is provided in Equation (5). The coefficients of the quadratic expressions (i.e., α and β) vary with the ratio of $\Pi_{3}$-to-$\Pi_{2}$. The coefficients and R-squared values that correspond to the curves shown in Figure 6 are provided in Table 3. The expressions are derived such that the ordinate intercept is set to zero.

Figure 7 indicates that $\Pi_{0}$ increases with respect to increases in $\Pi_{2}$. It follows that the critical buckling load, $P_{cr}$, increases with respect to increases in the radius of the helical members, until global buckling becomes the governing buckling mode. Once global buckling occurs, the curve flattens with respect to $\Pi_{2}$, as shown in the curve $b/r_{H}\;=\;100$ where $\Pi_{2}$ is approximately 0.015. As the *b*-to-$r_{L}$ ratio increases, the $\Pi_{0}$ vs. $\Pi_{2}$ curve shifts downward. The generalized quadratic expression that relates $\Pi_{2}$ to $\Pi_{0}$ is provided in Equation (6). The coefficients of the expression (i.e., α and β) vary with the ratio of $\Pi_{3}$-to-$\Pi_{1}$. The coefficients that correspond to the curves shown in Figure 7 are provided in Table 4. The expressions are derived such that the ordinate intercept is zero. The corresponding R-squared values are also provided in Table 4.

(5) $$\begin{array}{cc} \Pi_{0}= & \;\alpha \cdot \Pi_{1}^{2}+\beta \cdot \Pi_{1} \end{array}$$

(6) $$\begin{array}{cc} \Pi_{0}= & \;\alpha \cdot \Pi_{2}^{2}+\beta \cdot \Pi_{2} \end{array}$$

Figure 8 presents the interrelations of $\Pi_{0}$ and $\Pi_{3}$ for three values of the ratio $r_{L}$-to-$r_{H}$. As the $r_{L}$-to-$r_{H}$ ratio increases, the $\Pi_{0}$ vs. $\Pi_{3}$ curve shifts downward. The curve of best-fit that characterizes the trends between $\Pi_{0}$ and $\Pi_{3}$ is a power curve, provided in general terms in Equation (7). The coefficients, α and ξ, of the power curves vary with respect to the $r_{L}$-to-$r_{H}$ ratio. The coefficients are provided in Table 5 with the corresponding R-squared values.

(7) $$\begin{array}{cc} \Pi_{0}= & \;\alpha \cdot \Pi_{3}^{\xi } \end{array}$$

### 3.2. Analytical vs. FE Predictions of OLC

**Figure 9:**
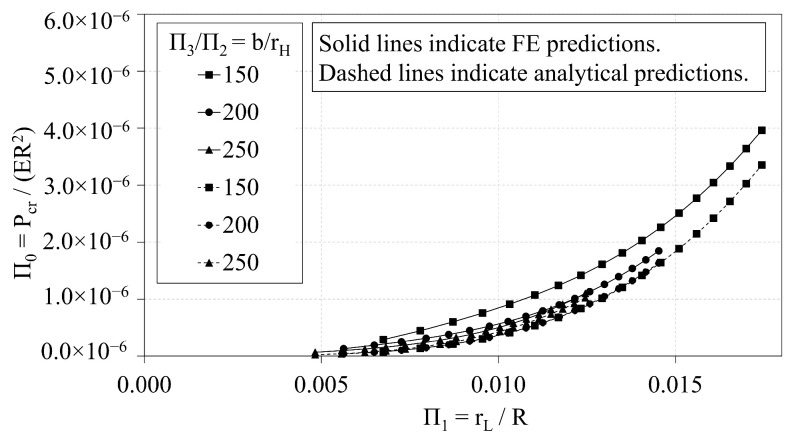
OLC $\Pi_{0}$ vs. $\Pi_{1}$ Analytical and FE predictions [26].

**Figure 10:**
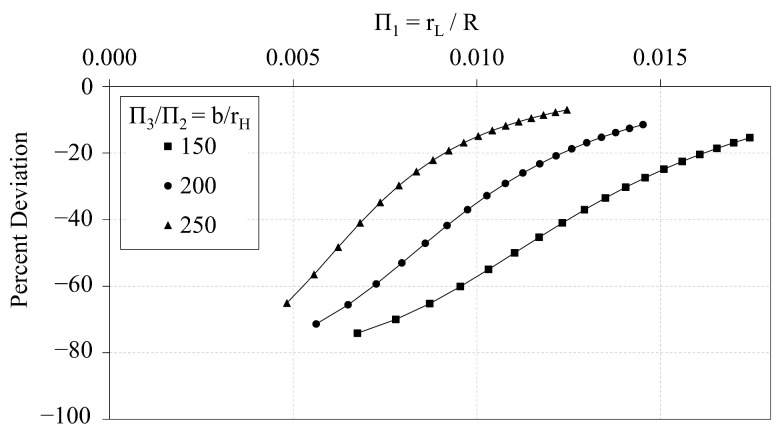
OLC $\Pi_{0}$ vs. $\Pi_{1}$ Percent deviation of analytical predictions from FE predictions [26].

**Figure 11:**
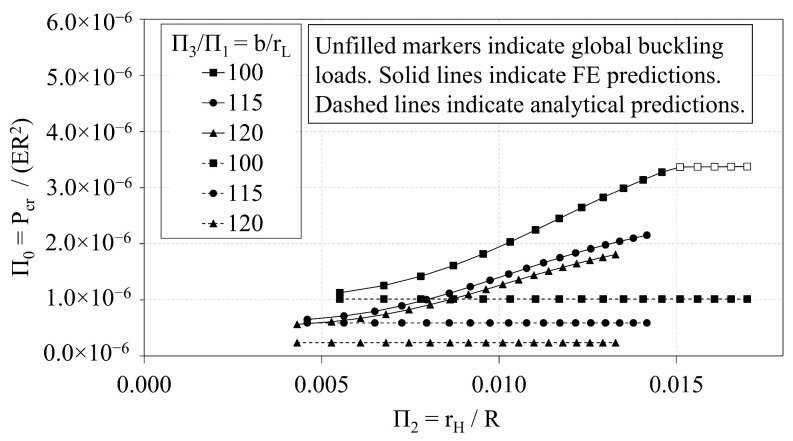
OLC $\Pi_{0}$ vs. $\Pi_{2}$ Analytical and FE predictions [26].

**Figure 12:**
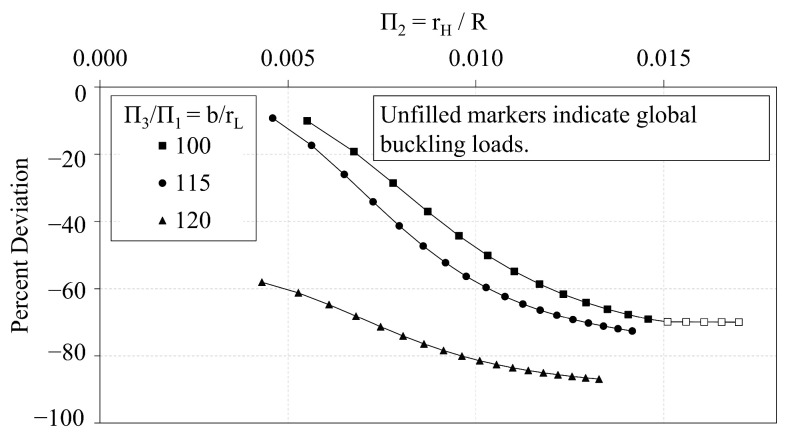
OLC $\Pi_{0}$ vs. $\Pi_{2}$ Percent deviation of analytical predictions from FE predictions [26].

**Figure 13:**
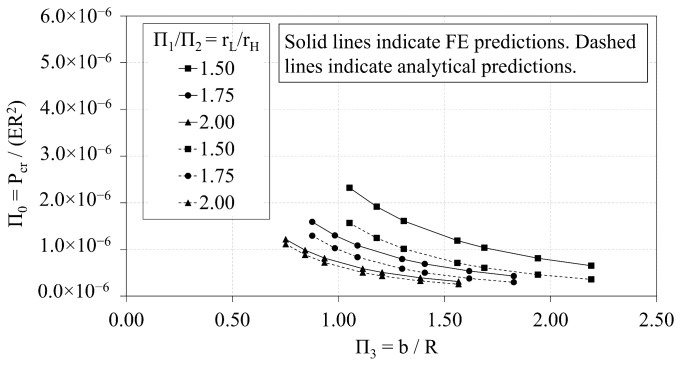
OLC $\Pi_{0}$ vs. $\Pi_{3}$ Analytical and FE predictions [26].

**Figure 14:**
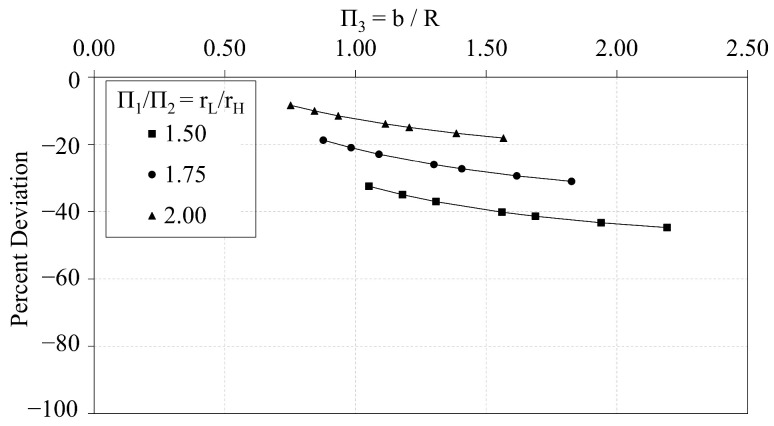
OLC $\Pi_{0}$ vs. $\Pi_{3}$ Percent deviation of analytical predictions from FE predictions [26].

In this section, the analytical predictions of the critical buckling loads of the OLC, $P_{cr_{anal}}$, are compared with the FE predictions, $P_{cr_{FE}}$. Figure 9 plots the analytical predictions and FE predictions of $\Pi_{0}$ vs. $\Pi_{1}$. The corresponding percent deviation (calculated via Equation (8)) of the analytical predictions with respect to the FE predictions is plotted against $\Pi_{1}$ in Figure 10. Similarly, Figure 11 and Figure 12 compare the predictions of $\Pi_{0}$ vs. $\Pi_{2}$ and illustrate the corresponding percent deviation, respectively; and, Figure 13 and Figure 14 compare the predictions of $\Pi_{0}$ vs. $\Pi_{3}$ and illustrate the corresponding percent deviation, respectively. Solid lines represent the FE predictions whereas the dashed lines indicate the analytical predictions.

(8) $$\begin{array}{c} \mathrm{Percent}\;\mathrm{Deviation}=\frac{P_{cr_{anal}}-P_{cr_{FE}}}{P_{cr_{FE}}}\cdot 100 \end{array}$$

### 3.3. Trend Analyses of OLC vs. ILC

**Figure 15:**
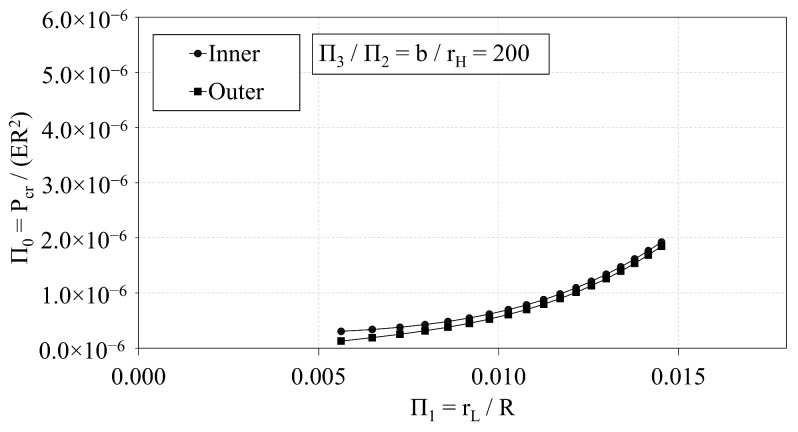
$\Pi_{0}$ vs. $\Pi_{1}$ of OLC and ILC [26].

**Figure 16:**
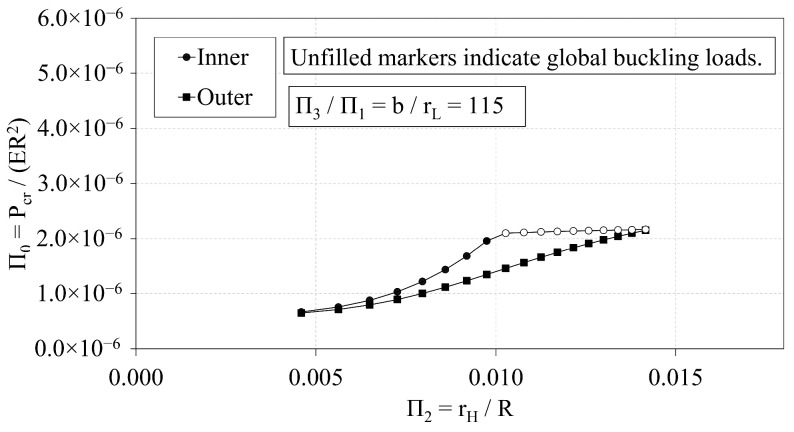
$\Pi_{0}$ vs. $\Pi_{2}$ of OLC and ILC [26].

**Figure 17:**
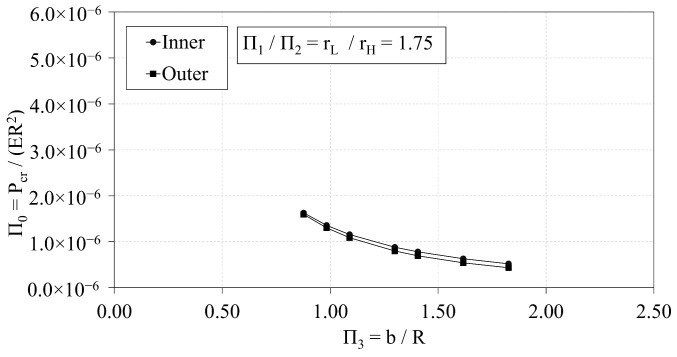
$\Pi_{0}$ vs. $\Pi_{3}$ of OLC and ILC [26].

In this section, the buckling capacity of the OLC and ILC are compared to assess the relative performance of the two configurations. The independent Π variables of the OLC and ILC Set 2 configurations are plotted with respect to $\Pi_{0}$ in Figure 15, Figure 16 and Figure 17.

Figure 15 demonstrates the interrelation of $\Pi_{0}$ and $\Pi_{1}$ of both the ILC and OLC, where the *b*-to-$r_{H}$ ratio is 200. Both configurations demonstrate a quadratic relation between $\Pi_{1}$ and $\Pi_{0}$. While the ILC curve indicates a greater buckling capacity than the corresponding OLC curve, the difference of the OLC curve relative to the ILC curve decreases dramatically from −60% to −4% as $\Pi_{1}$ increases from approximately 0.006 to 0.015.

Figure 16 demonstrates the interrelation of $\Pi_{0}$ and $\Pi_{2}$ of the ILC and OLC structures, where the *b*-to-$r_{L}$ ratio is 115. The plot once again demonstrates that the ILC possesses greater buckling capacity than the OLC for the Set 2 design space. As $r_{H}$ increases, the critical buckling load of the ILC increases quadratically, whereas the critical buckling load of the OLC increases more proportionally. At approximately $\Pi_{2}$ of 0.010, the ILC buckling mode transitions from local to global buckling, and the critical buckling load plateaus. Conversely, the OLC continues to be controlled by local buckling. This can be attributed to the placement of the longitudinal members at the outer diameter. With the longitudinal members at the outer diameter, the unbraced length of the longitudinal struts is increased compared to the ILC equivalent. In addition, the global moment of inertia of the OLC is greater than that of the ILC when both IsoTruss structures have the same number of nodes and outer radius (see Winkel [36]). Thus, the OLC is more susceptible to bay-level buckling and less susceptible to global buckling than the ILC equivalent. The difference of the OLC curve relative to the ILC curve decreases to less than −1% when $\Pi_{2}$ approaches 0.014.

Figure 17 demonstrates the interrelation of $\Pi_{0}$ and $\Pi_{3}$ of the ILC and OLC, where the $r_{L}$-to-$r_{H}$ ratio is approximately 1.75. The critical buckling load of the ILC once again exceeds that of the OLC in each case. The difference between the OLC design point and the ILC design point increases from −2% to −17% as $\Pi_{3}$ increases from 0.88 to 1.83.

### 3.4. Optimization of OLC vs. ILC

This final section incorporates the analytical expressions for the OLC in the gradient-based optimization routine. The OLC structure is optimized for mass with the same bounds as the ILC structure presented by Opdahl [26]. The constraints are also maintained the same, with the exception of local bay-level buckling. With the longitudinal members placed at the outer diameter of the structure, the OLC is not susceptible to shell-like buckling. The local bay buckling shown in Figure 4 is the same failure mode as longitudinal strut buckling in the OLC.

Table 6 presents the dimensions and mass of the optimized OLC and ILC structures. The optimized OLC has about 10.5% less mass than the optimized ILC for the prescribed constraints and bounds. The OLC optimum has more bays than the ILC optimum, however, the outer diameter of the OLC optimum is approximately 16% smaller than that of the ILC optimum. Even though the outer diameter has been reduced, the global moment of inertia is the same between structures. The Lagrange multipliers, $\lambda_{m}$, are shown with respect to the lower bound, upper bound, and structural failure constraints ($\lambda_{g}$, $\lambda_{sb}$, $\lambda_{l}$, and $\sigma_{u}$, respectively).

A local sensitivity analysis of each optimized configuration was performed by calculating the sensitivity derivatives of the mass and the constraints with respect to each design variable. The Jacobian matrices of the optimized OLC and ILC structures are presented in Equations (9) and (10), respectively.

(9) $$J_{OLC}=[\begin{array}{ccc} \frac{\partial M}{\partial N_{b}} & \frac{\partial M}{\partial N_{t_{L}}} & \frac{\partial M}{\partial D}\\ \frac{\partial \lambda_{g}}{\partial N_{b}} & \frac{\partial \lambda_{g}}{\partial N_{t_{L}}} & \frac{\partial \lambda_{g}}{\partial D}\\ \frac{\partial \lambda_{b}}{\partial N_{b}} & \frac{\partial \lambda_{b}}{\partial N_{t_{L}}} & \frac{\partial \lambda_{b}}{\partial D}\\ \frac{\partial \sigma }{\partial N_{b}} & \frac{\partial \sigma }{\partial N_{t_{L}}} & \frac{\partial \sigma }{\partial D} \end{array}]=[\begin{array}{ccc} 0.0177 & 0.0135 & 0.0205\\ 0 & -0.1000 & -0.4553\\ -4.018 & -0.2049 & 0\\ 0 & -0.0105 & 0 \end{array}]$$

(10) $$J_{ILC}=[\begin{array}{ccc} \frac{\partial M}{\partial N_{b}} & \frac{\partial M}{\partial N_{t_{L}}} & \frac{\partial M}{\partial D}\\ \frac{\partial \lambda_{g}}{\partial N_{b}} & \frac{\partial \lambda_{g}}{\partial N_{t_{L}}} & \frac{\partial \lambda_{g}}{\partial D}\\ \frac{\partial \lambda_{sb}}{\partial N_{b}} & \frac{\partial \lambda_{sb}}{\partial N_{t_{L}}} & \frac{\partial \lambda_{sb}}{\partial D}\\ \frac{\partial \lambda_{l}}{\partial N_{b}} & \frac{\partial \lambda_{l}}{\partial N_{t_{L}}} & \frac{\partial \lambda_{l}}{\partial D}\\ \frac{\partial \sigma }{\partial N_{b}} & \frac{\partial \sigma }{\partial N_{t_{L}}} & \frac{\partial \sigma }{\partial D} \end{array}]=[\begin{array}{ccc} 0.0215 & 0.0135 & 0.0184\\ 0 & -0.0834 & -0.3820\\ -0.508 & -0.191 & 0.0083\\ -4.50 & -1.69 & 0\\ 0 & -0.0073 & 0 \end{array}]$$

Row 1 of Equarions (9) and (10) indicate that the design variables of both configurations are positively correlated with the mass of the overall structures. Row 2 of Equarions (9) and (10) imply that the outer diameter has the greatest relative effect (i.e., inversely) on the global buckling load, while the number of bays is negligible. Row 3 of Equation (9) and Row 4 of Equation (10) indicate that the longitudinal buckling load of each configuration (manifest in the OLC as local bay-level buckling) is inversely related to the number of bays and number of longitudinal tows (i.e., positively correlated with the bay length). The sensitivity of the ILC with respect to the number of longitudinal tows is steeper than that of the OLC. Row 4 of Equation (9) and Row 5 of Equation (10) imply that the ultimate material stress is only affected by the number of longitudinal tows, and is inversely related. The sensitivity of the material stress of the OLC optimum is much steeper than that of the ILC optimum with respect to the number of longitudinal tows (for these particular optima).

## 4. Discussion

### 4.1. Influence of Helical Members

One of the most prominent themes from the analyses of the current study is the unprecedented contribution of the helical members to the critical buckling load of the OLC structures. The influence of the helical members can be assessed by the OLC interrelation curves, the plots comparing the analytical and FE predictions, and the percent deviation curves.

Figure 9, Figure 11 and Figure 13 demonstrate the extent to which the independent Π variables influence the dependent Π variable predicted from both analytical and FE predictions. The shapes of the curves shown in Figure 9 and Figure 13 indicate that the independent Π variables, $\Pi_{1}$ and $\Pi_{3}$, induce similar effects in $\Pi_{0}$ whether predicted using analytical or FE methods. Figure 9, conversely, indicates that changes in $\Pi_{2}$ affect the analytical and FE predictions differently. While the analytical predictions are not affected by changes in $\Pi_{2}$, the FE predictions indicate that increasing $\Pi_{2}$ increases the FE prediction of $\Pi_{0}$.

Likewise, the percent deviation curves indicate that the radius of the helical members has significant effect on the deviation between analytical and FE predictions. Figure 10 demonstrates that as the ratio $b/r_{H}$ decreases, the percent deviation curve is shifted downward, indicating an increase in the percent deviation. Thus, if all design parameters are fixed, and the helical radius is increased, the percent deviation will also increase. Figure 12 demonstrates that as $\Pi_{2}$ increases along each curve, the percent deviation also increases until global buckling is induced, at which point the critical buckling load is not changed with respect to the helical radius.

These results indicate that the analytical expression Equation (2) can be improved by incorporating the helical radius. One method would be to include the helical radius in the calculation of the boundary constraint coefficient, $\mu_{b}$. The analytical expressions for ILC strut buckling and ILC shell-like buckling both include derivations for boundary constraint coefficients as shown by Opdahl [26]. The strut buckling derivation calculates the flexural rigidity of the helical struts at the nodes, whereas the shell-like buckling derivation incorporates the bending energy from the intersecting helical members.

**Figure 18:**
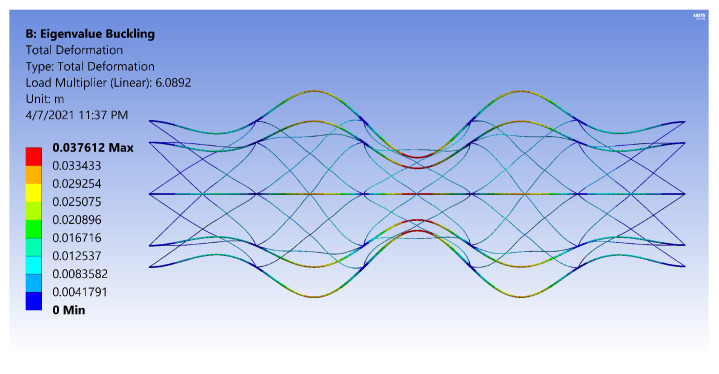
Local buckling of Set 2 OLC with two carbon tows in helical members [26].

**Figure 19:**
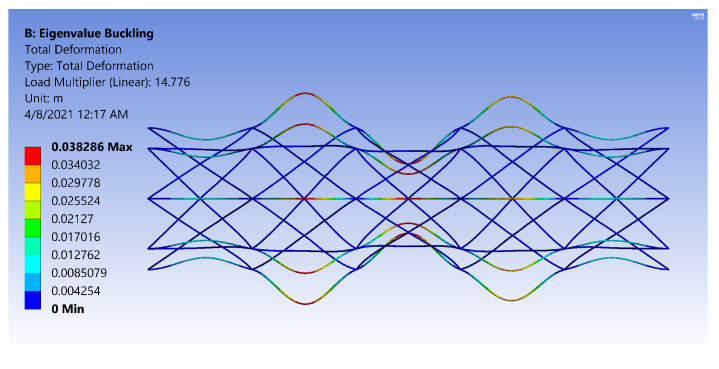
Local buckling of Set 2 OLC with thirteen carbon tows in helical members [26].

Figure 18 and Figure 19 are images produced from FE Models of OLC Set 2 structures. The figures have the same design parameters except the helical radius. Figure 18 has two carbon tows in the helical members, whereas Figure 19 has thirteen carbon tows in each helical member. By increasing the number of carbon tows, the rotation at the IsoTruss nodes is noticeably decreased, thereby increasing the flexural rigidity and localizing the deflection to the buckled longitudinal strut. The colors of the figures represent deflection.

**Figure 20:**
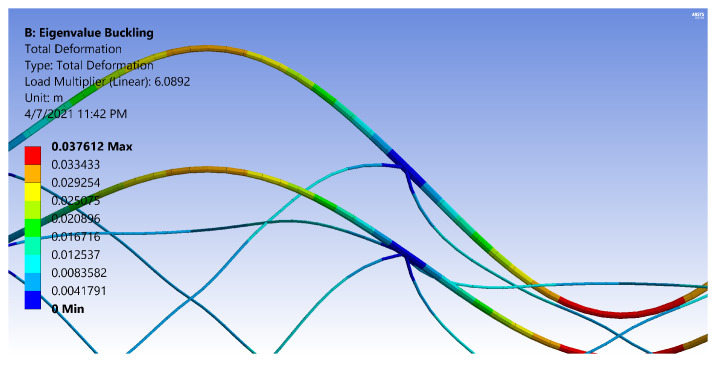
Rotation at the nodes of an IsoTruss structure with two carbon tows per helical member [26].

**Figure 21:**
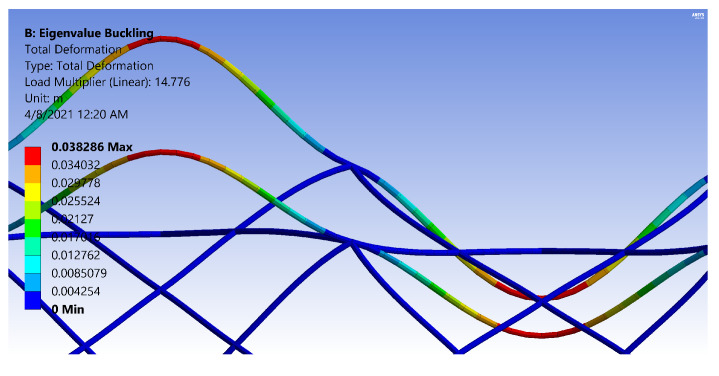
Rotation at the nodes of an IsoTruss structure with thirteen carbon tows per helical member [26].

While the boundary constraint of Figure 18 acts similar to a pinned connection, the boundary constraint of Figure 19 approaches the behavior of a fixed connection. The rotation of the helical constraints at the nodes of the OLC are magnified for clarity in Figure 20 and Figure 21.

Note that the helical members with two carbon tows (Figure 20) show enough rotation at the nodes to resemble a smooth inflection point, whereas the nodes of the thirteen-tow helical members (Figure 21) do not rotate as much, and flatten the longitudinal member at the nodes. Figure 20 and Figure 21 were reproduced with legends that indicate the total deformation corresponding to the buckling mode. The main purpose of the image is to indicate the reduced rotation at the nodes due to the increase in helical radius, hence, a color bar of the stress cloud diagram was not included in the current work. It is recommended that a boundary constraint coefficient be derived for bay buckling of OLC structures that incorporates the flexural rigidity demonstrated in the images.

### 4.2. OLC vs. ILC Performance

The relative performance of the OLC and ILC with respect to buckling is assessed from the comparative trend analyses and the optimization study. The trend analyses presented in Figure 9, Figure 11 and Figure 13 each demonstrate that the buckling capacities of the ILC structures exceed that of the corresponding OLC structures that possess the same outer radius, and are not independently optimized. Furthermore, Figure 11 demonstrates that the $\Pi_{0}$ of the ILC structure increases quadratically with respect to $\Pi_{2}$ until it transitions to global buckling. Conversely, the $\Pi_{0}$ of the OLC structure increases at a more shallow rate. The curves meet at approximately $\Pi_{2}=0.014$ where the OLC local buckling load corresponds with the ILC global buckling load.

While the design space of the trend analyses favored the ILC, the optimization analysis favored the OLC where both configurations were optimized with respect to mass. The optimized OLC has a shorter bay length, which increases the total mass due to the longer helical member length, but the OLC also has a smaller outer diameter and fewer longitudinal tows. The bottom line is that the OLC strength-to-weight exceeds that of the ILC, in part, by reducing the outer diameter. The outer diameter is approximately 16% smaller than the ILC configuration, and the overall weight is reduced by about 10.5%. The influence of the outer diameter in the ILC and OLC buckling behavior could have been manifest in the dimensional analysis if a trend analysis had been performed with respect to the outer diameter. One such analysis could be performed by plotting the Π variables $P_{cr}/\left(E\cdot r_{L}^{2}\right)$ versus $R/r_{L}$ where *R* varies for a fixed value of $r_{L}$.

## 5. Conclusions

The purpose of the current study is to characterize the buckling behavior of 8-node IsoTruss structures with outer longitudinal members. A dimensional analysis is performed to analyze the interrelations between the governing design parameters and the critical buckling load. The critical buckling loads of diverse geometric dimensions are predicted using finite element (FE) modeling in ANSYS WorkBench. The best-fit curves that indirectly relate the longitudinal radius, the helical radius, and the bay length to the critical buckling load are characterized as quadratic and power expressions. The FE predictions are also plotted with analytical predictions to assess the accuracy of the analytical expression for bay-level buckling with respect to FE methods. Changes in the longitudinal radius and the bay length induce similar trends in the FE and analytical predictions. Increasing the helical radius, however, does not induce the same trends in the analytical and FE predictions. While increasing the helical radius increases the FE prediction, there is no change in the prediction from the analytical expression.

Trend analyses are also performed on corresponding 8-node IsoTruss structures with inner longitudinal members. The buckling data of the inner longitudinal configurations (ILC) are plotted with the data of the outer configurations (OLC) to analyze the relative performance of the configurations with respect to buckling resistance. Each plot indicates that the ILC has greater buckling resistance than the outer longitudinal counter-part within the design space of the trend analysis where the dimensions of the ILC and OLC are equivalent. The relative performance of the OLC and ILC is also analyzed by optimizing both configurations with respect to mass. The optimized structures are subject to the same bounds, and the constraints are defined by analytical expressions that predict the relevant buckling modes of each configuration. The optimized OLC has about 10.5% less mass than that of the optimized ILC.

### Recommendations

First, a boundary constraint coefficient should be derived for the analytical expression that predicts local buckling in the OLC. The coefficient should incorporate the flexural rigidity of the helical members at the nodes, thereby capturing the effect of the helical radius on the buckling stability. Once derived, another trend analysis of $\Pi_{2}$ can be performed to determine if the analytical expression and FE model predict similar trends in the local buckling load by varying $r_{H}$. The improved analytical expression could be re-implemented in the gradient-based optimization code to improve the accuracy of the bay-level buckling constraint.

Second, additional research should be performed to delineate the design spaces where the ILC and OLC are preferred. While the results of the trend analyses indicate that the ILC has greater resistance to buckling than the OLC counter-part, the optimization analysis indicates that the optimized OLC has less mass than the optimized ILC. The advantage can be attributed to the fact that the OLC has a greater global moment of inertia than the ILC of equivalent outer radius. The design space could be delineated by performing a trend analysis with respect to the outer radius and the bay length.

## Figures and Tables

**Table 1:** Fixed Π Variables of Dimensional Analyses.

Π Variable	Set (1)	Set (2)	Set (3)
$\Pi_{1}$	$1.29\times 10^{-2}$	$1.13\times 10^{-2}$	$1.00\times 10^{-2}$
$\Pi_{2}$	$8.72\times 10^{-3}$	$6.50\times 10^{-3}$	$4.82\times 10^{-3}$
$\Pi_{3}$	1.31	1.30	1.21
$\Pi_{3}/\Pi_{2}$	150	200	250
$\Pi_{3}/\Pi_{1}$	100	115	120
$\Pi_{1}/\Pi_{2}$	1.5	1.75	2

**Table 2:** Fixed Design Parameters of FE Analyses.

Parameter	Units	Set (1)	Set (2)	Set (3)
$N_{t_{L}}$	N/A	11	12	13
$r_{L}$	[mm (in.)]	0.821 (0.0323)	0.859 (0.0338)	0.893 (0.0351)
$N_{t_{H}}$	N/A	5	4	3
$r_{H}$	[mm (in.)]	0.554 (0.0218)	0.495 (0.0195)	0.429 (0.0169)
*b*	[mm (in.)]	83.1 (3.27)	99.1 (3.90)	107 (4.22)
*R*	[mm (in.)]	63.5 (2.50)	76.2 (3.00)	88.9 (3.50)
$N_{b}$	N/A	30	25	23
*L*	[m (in.)]	2.49 (98.1)	2.48 (97.5)	2.47 (97.1)
$E_{z}$	[GPa ($10^{6}$ psi)]	161 (23.3)	161 (23.3)	161 (23.3)

**Table 3:** Coefficients relating $\Pi_{0}$ to $\Pi_{1}$.

$\Pi_{3}/\Pi_{2}$	α	β	R^2^
150	$1.81\times 10^{-2}$	$-1.02\times 10^{-4}$	0.99
200	$1.28\times 10^{-2}$	$-6.70\times 10^{-5}$	0.99
250	$1.11\times 10^{-2}$	$-5.59\times 10^{-5}$	0.98

**Table 4:** Coefficients relating $\Pi_{0}$ to $\Pi_{2}$.

$\Pi_{3}/\Pi_{1}$	α	β	R^2^
100	$5.60\times 10^{-3}$	$1.44\times 10^{-4}$	0.99
115	$3.71\times 10^{-3}$	$1.03\times 10^{-4}$	0.99
200	$3.68\times 10^{-3}$	$8.89\times 10^{-5}$	0.99

**Table 5:** Coefficients relating $\Pi_{0}$ to $\Pi_{3}$.

$\Pi_{1}/\Pi_{2}$	α	ξ	R^2^
1.5	$2.55\times 10^{-6}$	−1.73	1.00
1.75	$1.26\times 10^{-6}$	−1.78	1.00
2	$7.19\times 10^{-7}$	−1.85	1.00

**Table 6:** Results of OLC and ILC Multi-modal Optimization Analysis.

Property	OLC Minimum	ILC Local Minimum 1	OLC-to-ILC Ratio
Mass [kg (lb.)]	0.124 (0.273)	0.138 (0.305)	-
$N_{b}$	51	45	1.13
$N_{t_{L}}$	10	12	0.833
$N_{t_{H}}$	3	3	1.00
*D* [mm (in.)]	112 (4.39)	133 (5.24)	0.84
*b* [mm (in.)]	57.4 (2.26)	65.0 (2.56)	0.882
$I_{g}$ [cm^4^ (in.^4^)]	2.39 (5.75 $\times 10^{-2}$)	2.40 (5.76 $\times 10^{-2}$)	0.999
$\lambda_{m}$ (lower)	5.22 $\times 10^{-8}$	1.62 $\times 10^{-8}$	-
$\lambda_{m}$ (upper)	5.53 $\times 10^{-9}$	7.22 $\times 10^{-9}$	-
$\lambda_{m}$ (gb)	4.5 $\times 10^{-2}$	4.8 $\times 10^{-2}$	-
$\lambda_{m}$ (sb)	N/A	1.2 $\times 10^{-7}$	-
$\lambda_{m}$ (b and l)	8.2 $\times 10^{-7}$	2.2 $\times 10^{-9}$	-
$\lambda_{m}$ (σ)	6.3 $\times 10^{-14}$	6.3 $\times 10^{-14}$	-

## Data Availability

Some data, models, or code that support the findings of this study are available from the corresponding author upon reasonable request.

## References

[1] Vasiliev V.V., Barynin V.A., Razin A.F. (2012). Anisogrid composite lattice structures–development and aerospace applications. Compos. Struct..

[2] Yin S., Chen H., Wu Y., Li Y., Xu J. (2018). Introducing composite lattice core sandwich structure as an alternative proposal for engine hood. Compos. Struct..

[3] Jensen D., Strong B. (2002). The ultimate composite structure. Compos. Fabr..

[4] Rajak D.K., Pagar D.D., Menezes P.L., Linul E. (2019). Fiber-reinforced polymer composites: Manufacturing, properties, and applications. Polymers.

[5] Jensen D., Hinds K. Shear-Dominated Bending Behavior of Carbon/Epoxy Composite Lattice IsoBeam Structures. Proceedings of the 20th International Conference on Composite Materials.

[6] Francom L.R., Jensen D.W. (1999). Three-Dimensional Iso-Truss Structure. U.S. Patent.

[7] Hansen S.M. (2004). Influence of Consolidation and Interweaving on Compression Behavior of IsoTruss™ Structures. Master’s Thesis.

[8] Embley M.D. (2011). Damage Tolerance of Buckling-Critical Unidirectional Carbon, Glass, and Basalt Fiber Composites in Co-Cured Aramid Sleeves. Master’s Thesis.

[9] Umer R., Barsoum Z., Jishi H., Ushijima K., Cantwell W. (2018). Analysis of the compression behaviour of different composite lattice designs. J. Compos. Mater..

[10] Shitanaka A., Aoki T., Yokozeki T. (2019). Comparison of buckling loads of hyperboloidal and cylindrical lattice structures. Compos. Struct..

[11] Li M., Lai C., Zheng Q., Fan H. (2020). Multi-failure analyses of carbon fiber reinforced anisogrid lattice cylinders. Aerosp. Sci. Technol..

[12] Morozov E., Lopatin A., Nesterov V. (2011). Finite-element modelling and buckling analysis of anisogrid composite lattice cylindrical shells. Compos. Struct..

[13] Zheng Q., Jiang D., Huang C., Shang X., Ju S. (2015). Analysis of failure loads and optimal design of composite lattice cylinder under axial compression. Compos. Struct..

[14] Yoresta F.S., Nhut P.V., Matsumoto Y. (2020). Finite element analysis of axial compression steel members strengthened with unbonded CFRP laminates. Materials.

[15] Rozylo P., Ferdynus M., Debski H., Samborski S. (2020). Progressive failure analysis of thin-walled composite structures verified experimentally. Materials.

[16] Szklarek K., Gajewski J. (2020). Optimisation of the thin-walled composite structures in terms of critical buckling force. Materials.

[17] Bolton T. (2020). Buckling Analysis of Sandwich Composite Cylindrical-Conical Shells. Master’s Thesis.

[18] Doan Q.H., Thai D.K., Tran N.L. (2020). A numerical study of the effect of component dimensions on the critical buckling load of a GFRP composite strut under uniaxial compression. Materials.

[19] Genao F.Y., Kim J., Żur K.K. (2021). Nonlinear finite element analysis of temperature-dependent functionally graded porous micro-plates under thermal and mechanical loads. Compos. Struct..

[20] Maksimovic I.V., Maksimovic M., Maksimovic K. (2020). Stability and Initial Failure Analysis of Layered Composite Structures. International Conference of Experimental and Numerical Investigations and New Technologies.

[21] Ferreira F.P.V., Tsavdaridis K.D., Martins C.H., De Nardin S. (2021). Buckling and post-buckling analyses of composite cellular beams. Compos. Struct..

[22] Moita J.S., Araújo A.L., Correia V.F., Soares C.M.M., Herskovits J. (2020). Buckling behavior of composite and functionally graded material plates. Eur. J. Mech.-A/Solids.

[23] Li S., Qin J., Li C., Feng Y., Zhao X., Hu Y. (2020). Optimization and compressive behavior of composite 2-D lattice structure. Mech. Adv. Mater. Struct..

[24] Opdahl H.B., Jensen D.W. (2020). Validation of a Finite Element Model in ANSYS WorkBench for IsoTruss^®^ Structures in Uniaxial Compression. Utah NASA Space Grant Consortium.

[25] Veisi H., Farrokhabadi A. (2021). Investigation of the equivalent material properties and failure stress of the re-entrant composite lattice structures using an analytical model. Compos. Struct..

[26] Opdahl H.B. (2020). Investigation of IsoTruss Structures in Compression Using Numerical, Dimensional, and Optimization Methods. Master’s Thesis.

[27] McHale C., Hadjiloizi D.A., Telford R., Weaver P.M. (2020). Morphing composite cylindrical lattices: Enhanced modelling and experiments. J. Mech. Phys. Solids.

[28] Liu D., Lohse-Busch H., Toropov V., Hühne C., Armani U. (2016). Detailed design of a lattice composite fuselage structure by a mixed optimization method. Eng. Optim..

[29] Zhang L., Feih S., Daynes S., Wang Y., Wang M.Y., Wei J., Lu W.F. (2018). Buckling optimization of Kagome lattice cores with free-form trusses. Mater. Des..

[30] Belardi V., Fanelli P., Vivio F. (2018). Structural analysis and optimization of anisogrid composite lattice cylindrical shells. Compos. Part B Eng..

[31] Raouf N., Davar A., Pourtakdoust S.H. (2020). Reliability analysis of composite anisogrid lattice interstage structure. Mech. Based Des. Struct. Mach..

[32] Yang C., Hou X., Chang S. (2021). A synchronous placement and size-based multi-objective optimization method for heat dissipation design on antenna module of space solar power satellite. Sustain. Energy Technol. Assess..

[33] Yang C. (2021). An adaptive sensor placement algorithm for structural health monitoring based on multi-objective iterative optimization using weight factor updating. Mech. Syst. Signal Process..

[34] Rackliffe M.E., Jensen D.W., Lucas W.K. (2006). Local and global buckling of ultra-lightweight IsoTruss^®^ structures. Compos. Sci. Technol..

[35] McCune A.M. (2001). Tension and Compression of Carbon/Epoxy IsoTruss^TM^ Grid Structures. Master’s Thesis.

[36] Winkel L.D. (2001). Parametric Investigation of IsoTruss^TM^ Geometry Using Linear Finite Element Analysis. Master’s Thesis.

[37] Sui Q., Fan H., Lai C. (2015). Failure analysis of 1D lattice truss composite structure in uniaxial compression. Compos. Sci. Technol..

[38] Sui Q., Lai C., Fan H. (2017). Fundamental frequency of IsoTruss tubular composite structures. Arch. Appl. Mech..

[39] Opdahl H.B., Jensen D.W. (2021). Dimensional Analysis of Shell-like Buckling in IsoTruss® Structures using Numerical Methods. AIAA Scitech 2021 Forum.

[40] Kesler S.L. (2006). Consolidation and Interweaving of Composite Members by a Continuous Manufacturing Process. Master’s Thesis.

[41] Totaro G. (2012). Local buckling modelling of isogrid and anisogrid lattice cylindrical shells with triangular cells. Compos. Struct..

[42] Totaro G. (2013). Local buckling modelling of isogrid and anisogrid lattice cylindrical shells with hexagonal cells. Compos. Struct..

